# Prevalence of bovine trypanosomiasis in Côte d’Ivoire: Systematic review and meta-analysis

**DOI:** 10.4102/ojvr.v90i1.2069

**Published:** 2023-02-27

**Authors:** Jean-Yves Ekra, Edouard K. N’Goran, Léonard E.G. Mboera, Eliakunda M. Mafie

**Affiliations:** 1 SACIDS Foundation for One Health, Africa Centre of Excellence for Infectious Diseases of Humans and Animals in East and Southern Africa, Morogoro, Tanzania, United Republic of Tanzania; 2 Department of Microbiology, Parasitology and Biotechnology, College of Veterinary Medicine and Biomedical Sciences, Sokoine University of Agriculture, Morogoro, United Republic of Tanzania; 3 Département de Biochimie-Génétique, Unité de Formation et de Recherche (UFR) des Sciences Biologiques, Université Peleforo Gon Coulibaly, Korhogo, Côte d’Ivoire

**Keywords:** bovine trypanosomiasis, species, distribution, Cote d’Ivoire, meta-analysis, prevalence

## Abstract

Bovine trypanosomiasis is a parasitic disease caused by protozoans of the genus *Trypanosoma*. The disease cause economic losses in livestock production. In order to determine the status of research on this disease in Côte d’Ivoire, we used the systematic review method and meta-analysis. Three electronics databases, namely Google Scholar, PubMed and CrossRef were used to search for publications on trypanosomiasis prevalence that met our inclusion criteria. Twenty five articles were identified, 11 of which met the inclusion criteria. Bovine trypanosomiasis prevalence of 2.99% (95% confidence interval [CI]: 2.96% – 3.01%) to 25.28% (95% CI: 25.17% – 25.38%) were recorded between 1960 and 2021. The analyses showed that the most infected regions were the Bagoue 11.26% (95% CI: 11.25% – 11.27%), Bounkani 14.94% (95% CI: 14.93% – 14.95%), Gbeke 10.34% (95% CI: 10.33% – 10.35%), Marahoue 13.79% (95% CI: 13.78% – 13.80%), Poro 8.50% (95% CI: 8.49% – 8.51%), and Tchologo 11.83% (95% CI: 11.82% – 11.84%).The most sensitive diagnostic method used was the polymerase chain reaction (PCR). The species of trypanosomes diagnosed were *Typanosoma vivax* 4.99% (95% CI: 4.97% – 5.01%), *T. congolense* 1.51% (95% CI: 1.49% – 1.52%), and *T. brucei* 0.61% (95% CI: 0.59% – 0.62%). Despite some variation, the prevalence of bovine trypanosomiasis in Côte d’Ivoire caused mainly by *T. vivax* has increased in the years between 1977 and 2017. Efforts to control tsetse and other mechanical vectors should also be put in place to minimize its transmission.

**Contribution:**

The authors studied the prevalence of bovine trypanosomiasis using the systematic review method and MA in order to determine the status of research on this disease in Côte d’Ivoire.

## Introduction

Trypanosomes are flagellate unicellular protozoa belonging to the family Trypanosomatidae and the genus *Trypanosoma* (Aubry 2012). Many of the species that cause both animal and human trypanosomiasis belong to the genus *Trypanosoma* (Mekata et al. 2013). African animal and human trypanosomiasis are transmitted by tsetse flies of the genus *Glossina*. Nevertheless, some species have evolved to adapt to mechanical transmission, which has let them to spread beyond Africa’s tsetse zone, resulting in the ‘Nagana’ cattle wasting disease. Trypanosomes can be mechanically transmitted by hematophagous flies of the genera *Stomoxys* and *Tabanus* (Bafort, Kageruka & Timperman 1973). The ability of the parasites to remain in the vector’s mouth cavity is necessary for mechanical transmission. Therefore, the success of parasite transmission increases with the length of the blood-sucking gap of the vector between an infected and an uninfected animal (Desquesnes et al. 2013). The pathogens responsible for the most important trypanosomal infections in livestock are *Trypanosoma congolense* (Mackenzie et al. 1978) (savannah group), *Trypanosoma vivax* (Ziemann 1905) and *Trypanosoma brucei* (Difonzo & Bordia 1998). In Africa, trypanosomes are mainly found in the humid or subhumid areas (Sané et al. 2000) estimated to cover about 10 million km^2^. Trypanosomal infections in this zone constitute a major constraint to livestock development. Indeed, there are nearly 50 million cattle and about 70 million small ruminants continuously exposed to trypanosomiasis risk (Paylhard 2019; Fandamu et al. 2005). Typanosomiasis has a significant socio-economic impact and limits animal productivity in Africa with direct and indirect losses estimated at $4.5 billion (De La Rocque 2001, 2003).

Côte d’Ivoire is one of the most affected countries (Douati, Kupper & Kotia 1986). This is due to its vegetation, climate and hydrographic network which constitute an ecosystem favourable to the development of tsetse flies, the vectors of this disease. Despite current control measures, the prevalence of this disease is between 11% and 21% in the north of Côte d’Ivoire. The most affected areas are the departments of Korhogo, Boundiali and Ferkessédougou.

Despite its impact on the economy and animal health, bovine trypanosomiasis in Côte d’Ivoire has been disregarded in terms of control interventions (Sehi 2019). Furthermore, the military and political crises have devastated what little had been achieved in vector control. Although bovine trypanosomiasis has been studied in Côte d’Ivoire, the epidemiology of the disease remains poorly understood. In order to revitalise the fight against bovine trypanosomiasis, an inventory of the disease burden is critical.

In order to give a general overview of the epidemiology of bovine trypanosomiasis in Côte d’Ivoire, this systematic review and meta-analysis (MA) were conducted. Specifically, to analyse the trypanosomes species distribution; evaluate methods of detection and determine the prevalence of the disease.

## Materials and methods

### Search strategy and inclusion criteria

Following the guidelines in the Preferred Reporting Items for Systematic Reviews and Meta-Analyses (PRISMA) guide, the systematic review (SR) and MA were carried out (Moher et al. 2009). Inclusion and exclusion standards were established in accordance with the study’s objectives.

To find articles reporting the detection of trypanosome infection in cattle in Côte d’Ivoire between 1960 and 2021, a thorough search was performed. The databases of Google Scholar, PubMed and CrossRef were searched for relevant literature. The next search term was entered: The title, abstract and keywords, when appropriate, used the terms ‘bovine trypanosomiasis prevalence’ or ‘bovine trypanosomiasis and Côte d’Ivoire’. Regarding language and publication date, there were no limitations (the last search was performed on 17 July 2021). Also included were additional manual searches of author collections of pertinent peer-reviewed articles. For the purpose of choosing preliminary studies, Rayyan QCRI (Cambridge, United States and Doha, Qatar) (Ouzzani et al. 2016) was used. Articles from other databases were imported via Publish or Perish (Harzing 2007). Articles from PubMed were imported directly into Rayyan QCRI. References that were identical in terms of location, numerator, denominator and study period were eliminated. After a preliminary title and abstract screening, the complete texts of articles that were considered to be potentially relevant were examined. The bibliographies of the articles that were included were examined to locate additional pertinent and qualified articles.

### Inclusion and exclusion criteria

Relevant literature was searched, evaluated and analysed according to the PRISMA statement (Harzing 2007; Kamioka 2019). Inclusion and exclusion criteria, as well as search syntaxes, were predefined. Initially, references were selected based on their titles. Emphasis was placed on articles describing the epidemiology of bovine trypanosomiasis in Côte d’Ivoire, without limiting the date of publication. A previously stated inclusion criteria were established to include articles that offered relevant quantitative information on the epidemiology of bovine trypanosomiasis.

The references retained after the initial selection were analysed according to the abstract. Abstracts without full text, review articles, redundant information and studies that only describe the diagnosis of bovine trypanosomiasis were excluded. The full-text file of the publication was included if the data in the abstract were insufficient for evaluation. The following inclusion criteria were used to examine full-text publications: (1) A publication should contain information on cattle in Côte d’Ivoire; (2) contain information on the prevalence incidence and distribution of trypanosome species in cattle in Côte d’Ivoire.

The following causes contributed to the exclusion of full-text publications: (1) unspecified diagnostic test; (2) sample source not specified; (3) review articles; (4) articles reporting data published elsewhere; (5) cases reports based only on clinical signs; (6) epidemic reports without a lab confirmation and (7) reports of prevalence in any diagnostic test experimentation.

Publications with pertinent epidemiological data were included in the quantitative MA to determine the overall prevalence. Information such as cattle breeds, trypanosomes species responsible for the infections, sample size, method of diagnosis and prevalence were included in the analysis.

Prevalence estimation was performed after diagnostic tests categorisation. Thus, three categories were used to group the diagnostic tests: (1) parasitological methods such as wet blood smears, stained blood smears and microhematocrit concentration; (2) serological tests using antibody-based enzyme-linked immunosorbent assay (Ab-ELISA) and antigen-based enzyme-linked immunosorbent assay (Ag-ELISA) and (3) molecular tests including ordinary polymerase chain reaction (PCR) with various primers.

### Data extraction

Reference information such as author’s name, title and publication year were recorded in the database. The following data were extracted from the included publications: study area (department, region), periods of sample collection, cattle breeds, sample size, origin of the sample collected, diagnostic method used, number of positives, prevalence. Prevalence of trypanosomiasis was defined as the frequency of infection with *T. congolense, T. vivax* and *T. brucei* in a given population at a given time.

For articles that only reported the total number of animals sampled and the prevalence, the number of positives was estimated. Similarly, prevalence was estimated for article that reported only the number of samples and the number of positives. Case reports containing the required information, with the exception of prevalence data, were also included in the analysis. All data were recorded in an MS Excel spreadsheet.

### Statistical analysis

The data analysis was performed in several steps. Firstly, the mean prevalence was estimated by summing the number of cases in all studies included, divided by the total number of cattle sampled. If the year of data collection was not specified in the research, the publication year minus one (*n*−1) was considered. Secondly, the estimation of the total prevalence of bovine trypanosomiasis in the general sample size and their 95% confidence intervals (CIs) were evaluated by a random-effects. In these studies, heterogeneity was assessed by Cochran’s Q-test and the inverse of variance index (*I*). The variance index (*I*-squared) reflects the percentage of overall variation between studies due to heterogeneity and not by chance. *I*-squared values of 25%, 50% and 75%, respectively, indicate low, moderate and high degrees of heterogeneity. *I*-squared values of 0% imply that no heterogeneity was detected. On a standardised scale, *Q* is the weight of squares. It is presented as a *p*-value, with low values suggesting heterogeneity.

Subgroup analyses were carried out to identify potential sources of study heterogeneity. The study area (region and department), sample size, cattle breed, blood source, diagnostic method and trypanosome species were variables considered in the subgroup analysis. A funnel plot and an Egger plot allowed us to examine by visualisation the bias between studies. In addition, the statistical significance of bias was tested with Egger’s regression skewness test (Egger et al. 1997). Data were analysed using Comprehensive Meta-Analysis software version 3.3.070 (Biostat, Englewood, United States [US]). Using Quantum GIS version 2.0.1 (Open Source Geospatial Foundation, Boston, US) a map detailing the trypanosome species found at the research sites was developed.

### Ethical considerations

This article followed all ethical standards for research without direct contact with human or animal subjects.

## Results

### Literature selection

**FIGURE 1:**
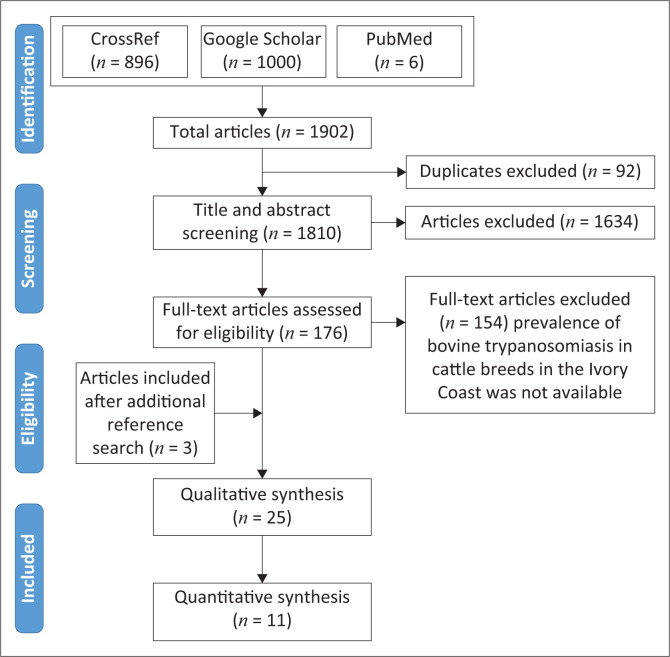
Preferred Reporting Items for Systematic Reviews and Meta-Analyses flow diagram of studies’ screening and selection.

A total of 1902 potential articles (Figure 1) were obtained from the literature searches. After reviewing titles, abstracts and excluding repeated articles from the database, for full text review, 176 articles were chosen. After examination of these articles based on our selection criteria, we obtained 25 articles that were eligible for systematic review. Of these 25 articles, 11 were selected to make the MA.

### Geographical distribution of bovine trypanosomiasis

The studies included in this review were conducted between 1979 and 2016 in 26 departments grouped into 12 regions (Bafing, Bagoue, Bere, Bounkani, Gkeke, Hambol, Kabadougou, Marahoue, Poro, Tchologo, Tompki and Worodougou). Sample sizes ranged from 87 to 3040 animals with infection prevalence ranging from 2.99 % (95% CI: 2.88% – 3.09%) to 25.28% (95% CI: 25.17% – 25.38%). With *I*-squared and *p*-value of 98.86 and *p* < 0.001, the prevalence of trypanosomiasis in cattle in Côte d’Ivoire was heterogeneous. Details of the studies are summarised in Table 1.

**TABLE 1:** Studies included in the quantitative synthesis.

References	Quality index score	Study periods	Diagnostics tests	Sample size	Positive case	Prevalence	s.e.	Variance	*Z-*value	*p*-value
Poivey, Camus & Landais 1983	10	1978–1979	Woo technique	2973	185	6.22	0.0759	0.006	−35.73	< 0.001
Acapovi-Yao et al. 2010	-	2002	ELISA	1470	44	2.99	0.1531	0.023	−22.725	< 0.001
Acapovi-Yao et al. 2016	-	March 2009 – April 2009	Woo technique	1270	171	13.5	0.0822	0.007	−22.632	< 0.001
Acapovi-Yao et al. 2015	-	-	Woo technique	1270	229	18.0	0.073	0.005	−20.746	< 0.001
Djakaridja et al. 2014	36	November 2011 – June 2012	Giemsa	660	25	3.78	0.2039	0.041	−15.864	< 0.001
Kouadio et al. 2014	12	July 2012 – August 2012	PCR	363	33	9.09	0.1825	0.033	−12.612	< 0.001
N’Djetchi et al. 2017	143	September 2013 – October 2013	PCR	87	22	25.28	0.2466	0.061	−4.392	< 0.001
Koffi et al. 2014	-	2014	PCR	200	41	20.5	0.1752	0.03	−7.738	< 0.001
Boka et al. 2019	10	July 2015 – October 2015	Giemsa	407	26	6.39	0.2027	0.041	−13.245	< 0.001
Yao et al. 2020	-	April 2016 – May 2016	Giemsa/PCR	582	22	3.78	0.2173	0.047	−14.893	< 0.001
Yéo et al. 2017	1	2017	Giemsa	360	34	9.44	0.1802	0.032	−12.543	< 0.001

Note: Heterogeneity of studies – *Q*-value = 318.683, *df* (Q) = 10, *p*-value = < 0.001, *I*-squared = 96.862.

s.e., standard error; PCR, polymerase chain reaction; ELISA, enzyme-linked immunosorbent assay.

**FIGURE 2:**
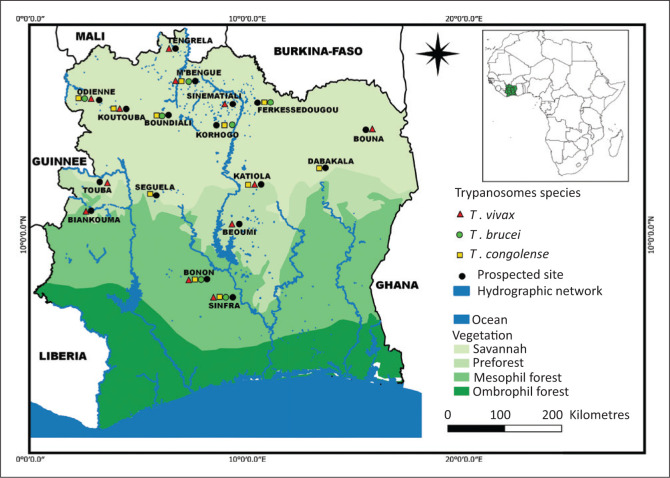
Distribution of the trypanosome species circulating in cattle in Côte d’Ivoire based on studies published between 1960 and 2021 included in the systematic review.

The spatial distribution of prevalence is illustrated in Figure 2, which shows that studies on the geographical distribution of bovine trypanosomiasis were mainly conducted in the northern, northwestern, northeastern and central regions of Côte d’Ivoire. The highest prevalence rates were observed in the departments of Bouna 14.94% (95% CI: 14.93% – 14.95%), Beoumi 14.68% (95% CI: 14.67% – 314.69%), Bonon 14.29% (95% CI: 14.28% – 14.29%), Sinfra 13.46% (95% CI: 13.45% – 13.47%), Boundiali 11.55% (95% CI: 11.54% – 11.56%), Ferkessedougou 11% (95% CI: 10.99% – 11.01%), Korhogo 8.69% (95% CI: 8.68% – 8.70%) and M’bemgue 8.36% (95% CI: 8.35% – 8.37%).

### Analysis and regression of data extracted from 11 studies included in the meta-analysis

The prevalence estimates of the 11 studies included in the MA are shown in Table 1-A1, Table 2-A1 and Table 3-A1. Heterogeneity was observed both at the level of the studies and in the subgroups defined as factors of variation. These subgroups or factors are region, department, method of diagnosis, breed of cattle and trypanosome species responsible for the infections. The subgroup analysis revealed large variations in prevalence and stable meta-regression models with *Z* = -11.13; *p* < 0.001.

The MA shows that the most infected regions were Bagouée, Bounkani, Gbeke, Marahoue, Poro and Tchologo with prevalence of 11.26% (95% CI: 11.25% – 11.27%), 14.94% (95% CI: 14.93% – 14.95%), 10.34% (95% CI: 10.33% – 10.35%), 13.79% (95% CI: 13.78% – 13.79%), 8.50% (95% CI: 8.49% – 8.51%) and 11.83% (95% CI: 11.82% – 11.84%), respectively. Through these analyses, the most sensitive diagnostic method was the PCR technique. Parasitological diagnostic techniques (Giemsa and Woo techniques) were more sensitive than ELISA test. These different techniques confirm the presence of trypanosome species such as *T. vivax, T. congolense* and *T. brucei*. The observed prevalence was 4.99% (95% CI: 4.97% – 5.01%), 1.51% (95% CI: 1.49% – 1.52%) and 0.61% (95% CI: 0.59% – 0.62%), respectively.

Regarding cattle breeds, the MA revealed that the least infected breed was the taurine breed Baoulé with a prevalence of 1.62% (95% CI: 1.60% – 1.64%). The taurine breeds (N’Dama and Baoulé) were less infected compared with the Zébu and Méré breeds whose prevalence rates were 3.67% (95% CI: 3.65% – 3.69%) and 3.50% (95% CI: 3.48% – 3.52%), respectively (Table 3-A1).

**FIGURE 3:**
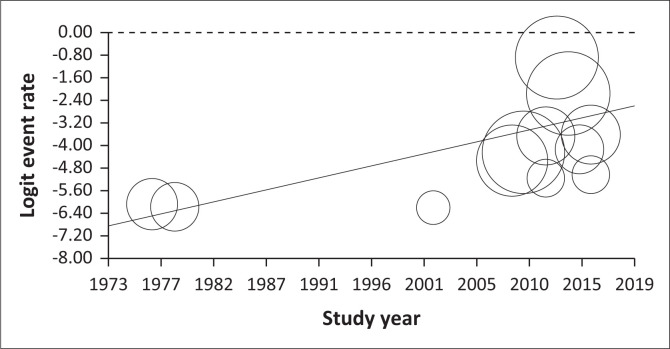
Prevalence regression by years of different studies.

**FIGURE 4:**
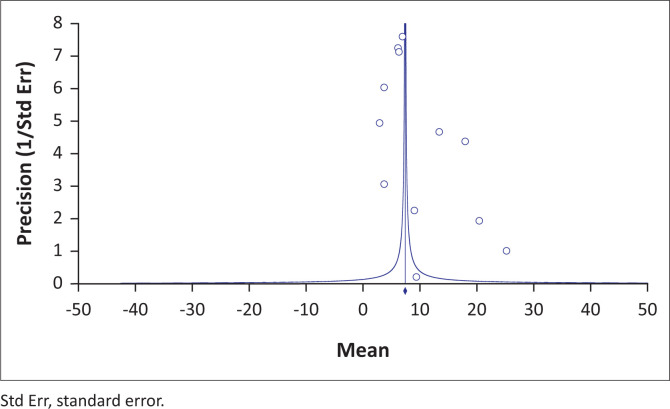
Plots of precision (1/standard error) by estimation of prevalence.

The meta-regression (Figure 3 and Figure 4) by survey year (considered as continuous variable) indicated a significant increase of the prevalence of bovine trypanosomiasis (*Z* = -11.13; *Q* = 191.05; degree of freedon [*df*] = 10; *p* < 0.001). Figure 3 and Figure 4 show the scatter plot of the regression of mean prevalence with survey year. The *p*-value for the survey year was < 0.05, indicating a stable pattern over time and space.

## Discussion

This study presents a systematic review of bovine trypanosomiasis prevalence and its pathogens in Côte d’Ivoire. The analyses indicate that bovine trypanosomiasis prevalence in Côte d’Ivoire has significantly increased over the years.

The increase in prevalence could be explained by several factors including limited number of studies and political prioritisation of the eradication of human African Trypanosomiasis over bovine trypanosomiasis. Since the first outbreak of the African trypanosomiasis, 11 studies over a period of 50 years have been conducted on bovine trypanosomiasis and the majority of these studies were concentrated in the last 18 years. Control and eradication of animal Trypanosomiasis have also been hampered by the various military-political crises in Côte d’Ivoire since 2000s. Tsetse fly control efforts have not been successful in these areas, unlike in areas endemic to human trypanosomiasis that were under state control during these crises (Kambiré et al. 2012).

The highest prevalence was found in the northern part of Côte d’Ivoire. These results are consistent with the findings of N’Djetchi et al. (2017). This zone is partly the major livestock area because of its vegetation and climate that favour livestock activity and in this area the animals have a role in the socio-cultural development and also a value (Sokouri et al. 2014). This territory is crossed by the four major rivers, which are the Bandama, Cavally, Sassandra and Comoé. These rivers are bordered by gallery forests (Acapovi-Yao et al. 2016; Camus 1981), which are known to be preferred sites for tsetse fly development (Bokar 1997; Sokouri et al. 2014). This area has several small dense forests, where cultural rites are conducted. These small forests are off-limits and constitute a favourable biotope for the proliferation of tsetse flies. The incriminated vectors are *Glossina palpalis palpalis* (Robineau-Desvoidy 1830), *G. palpalis gambiensis* (Vanderplanck 1949) and *G. tachninoides* (Westwood 1830) (Acapovi-Yao et al. 2010; Gouteux & Millet 1984). According to some authors (Djohan et al. 2015; Jamonneau et al. 2003), *G. palpalis gambiensis* and *G. tachninoides* are the vectors responsible for the transmission of animal trypanosomiasis. The high prevalence in the northern zone could be because of incorrect use of trypanocides, which promotes the development of trypanosome resistance (Simo et al. 2020). *Trypanosoma congolense, T. vivax* and *T. brucei* are commonly found in the 12 regions of Sudanese zone of Côte d’Ivoire (Solano et al. 1997) and are considered to be the most important pathogens in *Trypanosoma* infections in these regions (Acapovi-Yao et al. 2016; Poivey et al. 1983; Yao et al. 2020). Their presence may be related to the aforementioned specific tsetse species that are found in cattle in these farming areas mainly concentrated in the northern part of Côte d’Ivoire, which is the savannah zone.

The majority of studies included in this review have used parasitological methods. These methods are dependent on a high parasitemia in infected animals and on a morphological observation, which require expertise and experience of a technician making the observations (Hailu 2019). Irrespective of the technique used, PCR proved to be the most sensitive. The technique was validated using blood samples from experimentally infected animals, revealing its higher sensitivity and specificity when compared with parasitological techniques (Cox et al. 2005; Solano et al. 1999). This confirms the place of molecular techniques as the best means of diagnosis and identification of microorganisms.

The most predominant species of *Trypanosoma* was *T. vivax*. Similar observations have also been reported in Burkina Faso (Desquesnes & Dia 2003). This might be as a result of the transmission of *T. vivax* by other mechanical vectors as cattle are subjected to biting flies (Tabanids and *Stomoxys*) while grazing. According to Desquesnes et al. (2022), *T. vivax* geographical distribution is the whole of sub-Saharan Africa, the South and Central American and Iran. *T. congolense* savannah group is the agent responsible for the most severe form of animal Trypanosomiasis (Bengaly et al. 2002).

The analyses showed that the taurine breeds (indigenous) are less parasitized than Méré (cross-breed) and Zebu breeds. Other authors (Yao et al. 2020) reported similar results; this is because of an inherited trypanotolerance trait in taurines. Indeed, thanks to this genetic predisposition, these animals remain productive in these endemic areas. With the Méré, a breed resulting from cross-breeding taurines and zebu, the objective was to create a more hardy breed with this trypanotolerant trait. Unfortunately, high prevalence of trypanosomiasis among the Mérés suggests otherwise (Sokouri et al. 2009).

## Conclusion

This work represents the first attempt of a systematic review of bovine trypanosomiasis in Côte d’Ivoire. This review of systematic and MA provides comprehensive information on geographic distribution, infected cattle breeds and prevalence of bovine trypanosomiasis. The findings indicate variation in estimated prevalence depending on the diagnostic test used. Estimated bovine trypanosomiasis prevalence is high; however, variability was observed between departments and survey years. Based on the economic importance of the disease in livestock production, its detection in cattle is of great concern. It is recommended that PCR, which was the most sensitive technique, should be used in diagnosis and to facilitate control. Risk of transmission of trypanosomiasis in cattle in Côte d’Ivoire remain high. Considering the high prevalence of trypanosomiasis in crossbred cattle, crossbreeding in farms should be controlled. As *T. vivax* was one of the most important trypanosome species detected. In addition to regular tsetse control campaigns in the country, efforts to control other mechanical vectors should also be put in place.

## Appendix 1

**TABLE 1-A1:** Pooled prevalence estimates of bovine trypanosomiasis by region and department.

Number of studies	Region	Department	Sample size	Positive case	Prevalence	*Z*-value	*p*-value
1	Bafing	Touba	189	6	3.175	−8.238	< 0.001
4	Bagoue	Boundiali	1481	171	11.546	−25.041	< 0.001
		Tengrela	140	22	15.714	−7.233	< 0.001
		Kouto	69	0	0.000	−3.477	0.001
		Simpurgo	23	0	0.000	−2.694	0.007
1	Bere	Dianra	43	0	0.000	−3.140	0.002
		Mankono	48	0	0.000	−3.218	0.001
3	Bounkani	Bouna	388	58	14.948	−12.211	< 0.001
3	Gbeke	Beoumi	143	21	14.685	−7.447	< 0.001
		Bouake	60	0	0.000	−3.377	0.001
4	Hambol	Katiola	327	19	5.810	−11.784	< 0.001
		Dabakala	258	6	2.326	−9.048	< 0.001
5	Kabadougou	Odienne	2122	130	6.126	−30.151	< 0.001
		Korondougou	280	3	1.071	−7.796	< 0.001
		Koutouba	280	9	3.214	−10.049	< 0.001
1	Marahoue	Bonon	35	5	14.286	−3.709	< 0.001
		Sinfra	52	7	13.462	−4.580	< 0.001
9	Poro	Korhogo	4119	358	8.691	−42.522	< 0.001
		Sinematiali	131	6	4.580	−7.266	< 0.001
		Sirasso	23	0	0.000	−2.694	0.007
		M’bemgue	287	24	8.362	−11.228	< 0.001
7	Tchologo	Frkessédougou	2222	263	11.836	−30.577	< 0.001
2	Tonpki	Biankouma	143	6	4.196	−7.500	< 0.001
3	Worodougou	Kani	23	0	0.000	−2.694	0.007
		Seguela	193	6	3.109	−8.293	< 0.001

Note: Heterogeneity of department – *df* (Q) = 24, *p*-value = < 0.001, *I*-squared = 85.165.

*df*, degree of freedom.

**TABLE 2-A1:** Pooled prevalence estimates of bovine trypanosomiasis respect by diagnostic methods.

Number of studies	Blood origin	Diagnostics tests	Sample size	Positive case	Prevalence	*Z*-value	*p*-value
1	Jugular	ELISA	2940	44	1.497	−27.564	< 0.001
4	Ear	Giemsa	2009	107	5.326	−28.965	< 0.001
4	Ear	Woo-technique	8557	835	9.758	−61.060	< 0.001
3	Jugular	PCR technique	650	134	20.615	−13.906	< 0.001

Note: Heterogeneity – *Q*-value = 288.478, *df* (*Q*) 3= *p*-value = < 0.001, I-squared = 98.960.

PCR, polymerase chain reaction; ELISA, enzyme-linked immunosorbent assay; *df*, degree of freedom.

**TABLE 3-A1:** Pooled prevalence of bovine trypanosomiasis, stratified by bovine breed and trypanosome species.

Number of studies	Cattle breed	Sample size	Positive case	Prevalence	*Z*-value	*p*-value
2	Mere breed	4247	149	3.508	−39.740	< 0.001
2	Zebu breed	-	156	3.673	−40.045	< 0.001
2	N’Dama breed	-	116	2.731	−37.950	< 0.001
2	Baoule breed	-	69	1.625	−33.808	< 0.001
3	*T. congolense*	5563	84	1.510	−38.001	< 0.001
5	*T. vivax*	-	278	4.997	−47.860	< 0.001
3	*T. brucei*	-	34	0.611	−29.597	< 0.001

Note: Breed: Heterogeneity – *Q*-value = 38.120, *df* (*Q*) = 3, *p*-value = < 0.001, *I*-squared = 92.130. Trypanosome spp: Heterogeneity – *Q*-value = 201.403, *df* (*Q*) = 2, *p*-value = < 0.001, *I*-squared = 99.007.

T., *Trypanosoma; df*, degree of freedom.
